# Depression screening with patient-targeted feedback in cardiology: The cost-effectiveness of DEPSCREEN-INFO

**DOI:** 10.1371/journal.pone.0181021

**Published:** 2017-08-14

**Authors:** Christian Brettschneider, Sebastian Kohlmann, Benjamin Gierk, Bernd Löwe, Hans-Helmut König

**Affiliations:** 1 University Medical Center Hamburg-Eppendorf, Hamburg Center for Health Economics, Department of Health Economics and Health Services Research, Hamburg, Germany; 2 University Medical Center Hamburg-Eppendorf, Department of Psychosomatic Medicine and Psychotherapy and Schön Klinik Hamburg Eilbek, Hamburg, Germany; Mayo Clinic, UNITED STATES

## Abstract

**Background:**

Although depression is common in patients with heart disease, screening for depression is much debated. DEPSCREEN-INFO showed that a patient-targeted feedback in addition to screening results in lower depression level six months after screening. The purpose of this analysis was to perform a cost-effectiveness analysis of DEPSCREEN-INFO.

**Methods:**

Patients with coronary heart disease or arterial hypertension were included. Participants in both groups were screened for depression. Participants in the intervention group additionally received a patient-targeted feedback of their result and recommended treatment options. A cost-utility analysis using quality-adjusted life years (QALY) based on the EQ-5D was performed. The time horizon was 6 months. Resource utilization was assessed by a telephone interview. Multiple imputation using chained equations was used. Net-benefit regressions controlled for prognostic variables at baseline were performed to construct cost-effectiveness acceptability curves. Different sensitivity analyses were performed.

**Results:**

375 participants (intervention group: 155; control group: 220) were included at baseline. After 6 months, in the intervention group adjusted total costs were lower (-€2,098; SE: €1,717) and more QALY were gained (0.0067; SD: 0.0133); yet differences were not statistically significant. The probability of cost-effectiveness was around 80% independent of the willingness-to-pay (range: €0/QALY–€130,000/QALY). The results were robust.

**Conclusions:**

A patient-targeted feedback in addition to depression screening in cardiology is cost-effective with a high probability. This underpins the use of the patient-targeted feedbacks and the PHQ-9 that are both freely available and easy to implement in routine care.

## Introduction

Heart diseases and depression co-occur frequently. In their meta-analysis, Rutledge et al calculated that 21.5% of heart failure patients suffered from a co-morbid clinically significant major depression [1]. Similar results were reported by Thombs et al for survivors of acute myocardial infarction of whom 19.8% suffered from depression [2]. Nicholson, Kuper and Hemingway showed that patients with depression have a 80% higher risk of developing a coronary heart disease [3]. The co-existence of heart diseases and major depressive disorder is associated with higher mortality, increased health care utilization, higher risks of hospitalization, and decreased health related quality of life (HRQL) [1,4–7].

Screening and early diagnosis of a comorbid depression is recommended by different agencies. The British National Institute for Health and Care Excellence (NICE) recommends case identification and recognition in patients with chronic physical health problems [8], whereas the American Heart Association (AHA) specifically recommends depression screening for all patients suffering from chronic heart disease (CHD) [9]. However, there are no randomized controlled trials assessing the efficacy of depression screening in patients with heart diseases [10,11]. Evidence was exclusively derived from observational studies, e.g. Burton et al, reporting a small increase in the number of new diagnoses and treatments [11,12].

Apart from the physician gaining information about the depression status of his/her patient, the patient himself/herself could benefit from the screening by gaining some insight into his/her mental health status. This effect remained widely unconsidered in previous studies. The DEPSCREEN-INFO (Increasing the Efficiency of Depression-screening Using Patient-targeted Feedback) trial was designed to investigate the efficacy of a patient-targeted feedback after screening. The analysis of efficacy showed a significant improvement in depression severity (measured by the Patient Health Questionnaire-9 (PHQ-9)) over a period of six months [13].

The proof of efficacy is the first and necessary step in the process of health technology assessment. However, due to the scarcity of resources in health care, policy makers also require information on cost-effectiveness to make decisions for the benefit of all stakeholders in the health care system.

In this article we present the economic evaluation of the DEPSCREEN-INFO trial. The purpose was to perform a cost-effectiveness analysis of patient-targeted feedback after screening in cardiac patients in comparison to screening alone from a societal perspective in Germany.

## Methods

### Sample

**Fig 1:**
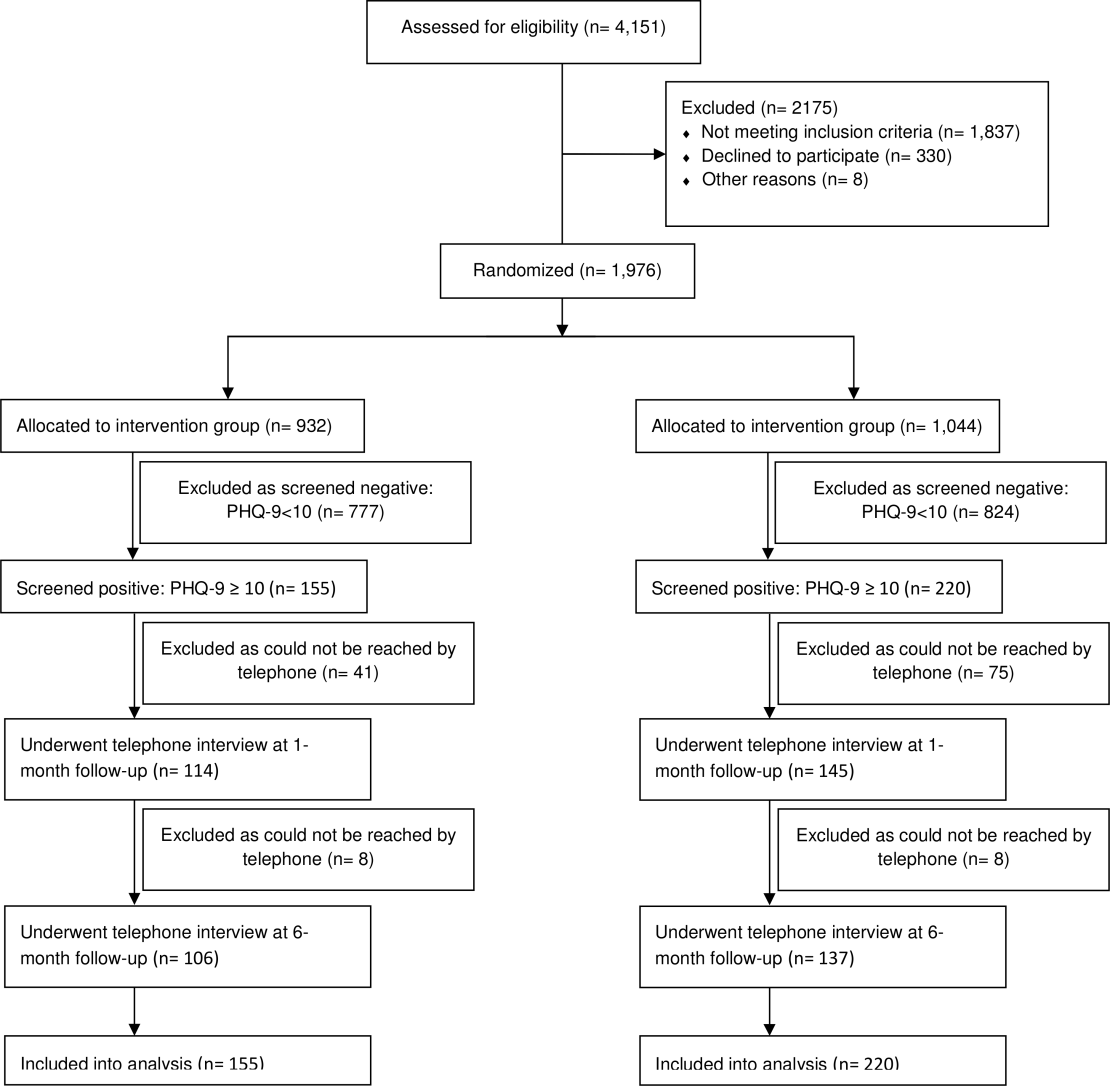
CONSORT flow diagram.

DEPSCREEN-INFO (German Clinical Trials Register: DRKS00003277; ClinicalTrials.gov: NCT01879111) is an observer-blinded randomized controlled trial focussing on the efficacy of patient-targeted feedback after depression screening in terms of a decrease of depression severity. The methods have been described in detail elsewhere [13]. Study participants were recruited between October 2011 and October 2013 from two cardiology outpatient centers and one cardiology inpatient ward in Hamburg, Germany. Details about the recruitment process and the study course can be found in the CONSORT flow diagram (Fig 1). Participants were included if they had a clinical diagnosis of coronary heart disease or arterial hypertension, were 18 years or older, and had sufficient German language skills. Participants were excluded if they had an acute life-threatening condition, severe somatic or psychiatric disorders with immediate necessity of professional intervention, active suicidality, and severe cognitive or visual impairment. Participants were randomized either to depression screening plus patient-targeted feedback (Intervention group (IG)) or depression screening alone (Control group (CG)) in a 1:1 ratio by coin toss. Due to the nature of the intervention the blinding of patients was not possible. Investigators and outcome raters were blinded. The study sample consisted of 375 patients. 155 patients were included in the IG and 220 in the CG.

### Intervention

Patients in IG and CG were recruited and screened during the waiting time before an outpatient appointment or in the inpatient wards by means of the self-reported version of the PHQ-9 [14]. Depression screening was conducted according to the current AHA guidelines [9]. Patients were screened positive if they reached a score of 10 or higher on the PHQ-9, i.e. at least moderately depressed. In this case the treating physician received a written feedback of the screening result before the consultation with the patient. The written feedback indicated the severity of depression using the pictogram of a traffic light (orange light = moderately depressed; red light = severely depressed) and described the corresponding guideline-based treatment [15]. The treating physician decided whether to use this information in the process of treatment. This applied to IG and CG. In the IG all participants additionally received a patient-targeted feedback consisting of a patient feedback form indicating the severity level of depression as well as a brief explanation regarding its clinical relevance and -in case of a positive screening result- a 2-page written patient information sheet regarding guideline-based depression diagnosis and treatment [15] (http://www.patienten-information.de/mdb/downloads/kip/aezq-version-kip-depression.pdf) as well as the contact information of the local university psychosomatic outpatient clinic.

### Data collection and measures

#### Data collection

The data collection process has been described in detail elsewhere [13]. In brief, all study participants were assessed at baseline (T0) by a self-reported questionnaire. After 1 month (T1) and after 6 months (T2) structured telephone interviews were conducted. Besides sociodemographic information (age, gender, living situation, years of education), the symptom severity of angina pectoris (Canadian Cardiovascular Society (CCS) grading of angina pectoris) [16], the symptom severity of depression (PHQ-9) [14] and the perceived mental illness stigma (Stig-9) were assessed. As we planned to perform the economic evaluation from the societal perspective we calculated quality-adjusted life years and used the EQ-5D-3L as measure of HRQL [17]. Fatalities were recorded either in the course of the telephone interviews at follow-up or -in case that the follow-up interview could not be conducted- by means of the registers of birth, marriage and deaths which are statutorily organized by the German civil registry offices. Service utilization was assessed by a modified German version of the Client Sociodemographic and Service Receipt Inventory (CSSRI) [18]. The dataset can be found at figshare (10.6084/m9.figshare.5203633)

#### EQ–5D-3L

The EQ–5D-3L is the three level version of a generic health-related quality of life (HRQL) questionnaire that consists of five questions (items) assessing current problems in the dimensions: mobility; self-care; usual activities; pain/discomfort; and anxiety/depression [17]. The possible answers for each question are coded as follows: 1, no problems; 2, moderate problems; 3, extreme problems. Based on this the EQ-5D descriptive system is able to describe 243 (3^5^) health states, e.g. 11223 describes a health state in which the patient has no problems in mobility (1) and self-care (1), moderate problems in the performance of usual activities (2), moderate pain or discomfort (2) and severe problems caused by anxiety or depression (3).

The EQ–5D also includes a visual analogue scale (EQ VAS), which is similar to a thermometer, ranging from 0 (worst imaginable health state) to 100 (best imaginable health state). The EQ VAS describes the respondent’s self-rated valuation of his/her health state.

The EQ-5D has been validated successfully in populations with heart diseases and populations with depression [19–22]. Furthermore, it is possible to calculate a utility score (EQ-5D index score) based on the EQ-5D descriptive system. These utility scores were derived from surveys of the general population and represent preferences of the general population for the different health states of the EQ-5D descriptive system. In the present study EQ-5D index scores from the UK [23] were used that were derived from a large general population sample (n = 2,997). These EQ-5D index scores range from -0.594 (worst health state) to 1 (best health state).

#### Questionnaire of service utilization and costs

Service use and costs were measured from the societal perspective. The questionnaires covered the period of the last month at T1 and the period of the last 5 months at T2. In the assessment of direct costs inpatient services, outpatient physician services, outpatient non-physician services (e.g. physiotherapy, occupational therapy, exercise therapy, massage), outpatient psychotherapist services, medication, ambulatory nursing care and informal care were considered. Depending on the service, quantity of use or duration was recorded (Table 1). Additionally, indirect costs due to productivity losses were assessed for paid work (absenteeism). Reduced productivity at work (presenteeism) was not included.

**Table 1:** Cost categories and sources of applied unit costs.

Sector	Service / Goods	Units	Monetary values (unit costs)
Inpatient services	General hospitals, psychiatric hospitals and hospitals for rehabilitation	Days	Type specific mean rates [24]
Outpatient physician services	GP, specialists (e.g. cardiologist, internist, ophthalmologist)	Contacts	Type specific mean rates[24]
Outpatient non-physician services	e.g. physiotherapy, massage, lymph drainage, ergotherapy	Contacts	Reimbursement schedule[24]
Outpatient psychotherapist services	Psychotherapist	Contacts	Reimbursement schedule[24]
Medication	Product	Quantity	Official pharmaceutical index (Rote Liste) [25]
Nursing care	Ambulatory nursing care	Hours	Type specific wage[24]
	Informal care	Hours	Type specific wage (replacement cost approach)[24]
Indirect costs	Productivity losses	Hours	Gross income plus nonwage labor costs [26]

#### Unit costs

Unit costs from the societal perspective were applied to all privately purchased or prescribed services and goods. Costs were calculated in EUR at 2012 price level. Costs were not discounted as of the time horizon was only 6 months.

Detailed information regarding the unit costs is shown in Table 1. Unit costs were mainly extracted from the German standardised unit cost catalogue by Bock et al [24]. The only exception was medication. The valuation of medication was based on drug codes, dosage and duration and was valued by means of the German pharmaceuticals database `Rote Liste´ [25]. Costs for inpatient services were calculated using average costs per day differentiated by hospital type (general hospital, psychiatric hospital, rehabilitation hospital). Outpatient physician services and outpatient psychotherapist services were valued by means of average costs per contact. Outpatient non-physician services measured in contacts were calculated based on reimbursement schemes of the German statutory sickness funds. Ambulatory nursing care measured in hours was valued using the reimbursement schemes of the German statutory sickness funds. Informal care was valued using the replacement cost method. This means it was assumed that informal care could have been substituted by paying a professional caregiver. Therefore hours of informal care were valued using the same hourly wage rate as for the valuation of workers in the commercial sector `Social care for older adults and challenged persons´[24]. Indirect costs were valued based on the human capital approach by using gross income plus nonwage labor costs [26]. Total costs after 6 months were calculated as sum of direct and indirect costs at T1 and T2.

#### Intervention costs

Intervention costs were not incorporated in the analysis as the difference between IG and CG resulting from the intervention was very small. Patients in the IG were only provided with a patient feedback form, a 2-page patient information sheet regarding guideline-based depression diagnosis and treatment, and the contact information of the local university psychosomatic outpatient clinic.

#### Calculation of QALY

We measured the EQ-5D at T0, T1 and T2. It was assumed that the development of quality of life between two time points is subjected to a linear trend. As several participants had effective observation periods deviating from the planned periods, there was a risk of an overestimation of the gain/loss of quality of life. Therefore, we adjusted the QALY for the effective observation period. The following equations were used to calculate QALY for the period from T0 to T1 (1 month) and for the period from T1 to T2 (5 months):

$${QALY}_{T1}=\left[{Index}_{T0}+[\frac{\left({Index}_{T1}-{Index}_{T0}\right)}{2}]*[\frac{30}{days\;(T0;T1))}]\right]*\left(\frac{1}{12}\right)$$

$${QALY}_{T2}=\left[{Index}_{T1}+[\frac{\left({Index}_{T2}-{Index}_{T1}\right)}{2}]*[\frac{150}{days\;(T1;T2))}]\right]*\left(\frac{5}{12}\right)$$

No patient died between baseline and T1. For patients who died between T1 and T2 the date of death was not retrievable for all participants. Therefore, we treated all deceased participants as died at T1. The same procedure was applied in the calculation of costs of deceased participants. QALY after 6 months were calculated by adding up QALY at T1 and QALY at T2. QALY were not discounted as the time horizon was only 6 months.

### Statistical analysis

Missing values were a frequent phenomenon in the DEPSCREEN-INFO trial. In total 135 variables were considered in the study. The proportion of missing values in the considered variables ranged from 0% to 30% (IG) and 35% (CG). 54% of the participants (IG: 57%; CG: 51%) had no missing values. 18% of the participants (IG: 17%; CG: 19%) gave no information at T1 and 26% of the participants (IG: 23%; CG: 28%) gave no information at T2. Missing values in variables assessing resource utilization and clinical parameters were imputed on item level by multiple imputation using chained equations (MICE) [27–29]. We created 100 imputation based on data assessed at baseline as well at T1 and T2. Imputation of incomplete variables was performed under fully conditional specification [30]. Missing values were imputed by the “mi” command of Stata 14 [31]. Calculation of costs and QALY was performed after completion of the imputation procedures. Multiple imputation resulted in 100 data sets with N = 375 participants per data set.

#### Base case analysis

All analyses were performed by means of the “mi estimate” command of Stata 14 [31]. This means that in a first step parameters model parameters were estimated for each imputed dataset specifically by the statistical approaches described beneath. In a second step these estimates along with their standard errors were combined to one estimate and standard error using Rubin´s rule [32].

Sample characteristics at baseline and differences in unadjusted mean costs and unadjusted mean QALY in the time periods between baseline and T1, between T1 and T2 and over the complete study period of 6 months were analysed using linear and logistic regression, respectively. Mean costs and mean QALY in the complete study period of 6 months were adjusted for age, gender, years of schooling, symptom severity of angina pectoris at baseline, baseline HRQL and the perceived mental illness stigma at baseline. Adjustment was performed by using linear regression.

The analysis of cost-effectiveness after 6 months was based on calculation of the incremental cost-effectiveness ratio (ICER). The ICER is the ratio of the differences in mean total costs $\bar{C}$ and mean total health effects $\bar{E}$ (quality-adjusted life years (QALYs) [33]) between IG and CG:

$$ICER=\frac{{\bar{C}}_{IG}-{\bar{C}}_{CG}}{{\bar{E}}_{IG}-{\bar{E}}_{CG}}=\frac{\Delta \bar{C}}{\Delta \bar{E}}$$

As the ICER is a point estimate it does not consider uncertainty in the data. Therefore, we constructed a cost-effectiveness acceptability curve by means of a series of net benefit regressions (NBR) using different willingness-to-pay (WTP) margins [34]: An intervention is considered cost-effective, if

$$\bar{NB}=\Delta \bar{E}\times \lambda -\Delta \bar{C}>0$$

where $\bar{NB}$ is the net monetary benefit and *λ* is the willingness-to-pay for one QALY. Hence, $\bar{NB}$ is a transformation of the ICER under consideration of WTP for one QALY and a simple indicator of cost-effectiveness and does not indicate statistically significant results. Relevant for further analyses is the patient-specific net benefit *NB*_*i*_ = *E*_*i*_ × *λ* − *C*_*i*_, which can be used to model the uncertainty and variability in the data. It was analyzed as dependent variable by multiple regression analysis (NBR):

$$NB_{i}{}_{.}=\alpha +\beta g_{i}+\varepsilon_{i}$$

where the constant *α* is the mean ${\bar{NB}}_{i}$ in the CG, *g* the dummy variable for the treatment group (1 = IG, 0 = CG), *β* is the mean incremental net benefit ${\bar{NB}}_{IG}-{\bar{NB}}_{CG}$ of the intervention compared to the control group and *ε*_*i*_ is the stochastic error term. NB_i_ was adjusted for age, gender, years of schooling, symptom severity of angina pectoris at baseline, baseline HRQL, and perceived mental illness stigma at baseline by adding these variables as covariates in NBR. For constructing CEACs we applied the net-benefit approach [35–37]. Probabilities of cost-effectiveness based on repeated regression analyses using different values for *λ* were plotted on the y-axis against values for *λ* on the x-axis. The probability of cost-effectiveness corresponds to 0.5 x p-value of the regression coefficient *β*, if *β* < 0, or 1–0.5 x p-value, if *β*>0. A rationale for the use of the p-value can be found in the papers by Hoch et al. [35,36]. In summary, the p-value of the treatment group dummy represents the statistical uncertainty of cost and effect data. A positive coefficient of the treatment group dummy indicates a positive $\bar{NB}$ and cost-effectiveness at a specific WTP margin, a negative coefficient indicates a negative $\bar{NB}$ and the lack of cost-effectiveness. Furthermore, it is necessary to multiply the p-value by 0.5 as the CEAC can be described as a one-sided test. For *λ* we chose a range from €0 per QALY to €130,000. €130,000 per QALY represents the updated willingness-to-pay margin for acceptable cost-effectiveness (formerly 50,000 per QALY) [38]. The NBR were performed by linear regressions.

#### Sensitivity analyses

To assess the influence of the mode of QALY calculation on the results we additionally performed all analyses based on QALY calculated by means of the EQ VAS as a measure of HRQL (Utility weight = EQ VAS score / 100).

As the number of deceased patients was higher in the CG than in the IG the decision to treat all patients deceased between T1 and T2 as deceased at T1 could be a disadvantage for the CG. Therefore, in an additional sensitivity analysis we only included deceased patients from the IG and treated all patients from the CG as survivors.

To analyse the intervention from a health care payer perspective we performed a further analysis excluding costs from informal care and productivity losses.

As described above we adjusted QALY to the planed observation periods assuming a linear development. To describe the effect of this approach we performed the analyses based on not adjusted QALY.

All statistical analyses were carried out by using STATA 14 (Stata Corp., College Station, TX, USA).

### Ethics

Written informed consent was obtained before participants were included. The study was approved by the ethics committee of the Medical Association, Hamburg, Germany.

## Results

### Characteristics of the study population at baseline

The mean age was 63.28 years (SD: 11.72; Range: 27–91). More men than women participated in the study (male: 58%; female: 42%). 39% of the participants were living alone. The mean symptom severity of angina pectoris was moderate. 38% of patients had no symptoms, 20% had limitations during strenuous physical activity, 19% had limitations during light physical activity and 23% had limitations independent from physical activity. HRQL measured by the EQ-5D index and the EQ VAS was notably reduced. The mean score of the EQ VAS was approximately 15 point lower than in the German general population (age group: 55 and 74 years) [39]. The mean severity of depression was moderate (mean PHQ-9 score: 13.6; SD: 3.3). There were no statistically significant differences between IG and CG in baseline characteristics (Table 2).

**Table 2:** Baseline characteristics and group comparsion of the imputed sample (m = 100).

Characteristics		Intervention group	Control group	p-value
Age (years)				0.27^a^
	mean (SD)	62.5 (11.4)	63.8 (11.9)	
Female: %		46.5%	39.1%	0.16^b^
Living situation: %				0.33^b^
	Alone	41.6%	36.5%	
	With another person	58.4%	63.5%	
Years of education: %				0.82^b^
	Less than 10 years	47.0%	48.3%	
	10 years or more	53.0%	51.7%	
CCSC grade at baseline: %				
	0	32.6%	42.4%	
	1	18.6%	20.9%	0.63^b^
	2	23.2%	15.3%	0.02^b^
	3	25.6%	21.4%	0.12^b^
EQ-5D index (-0.59–1)				0.11^a^
	mean (SD)	0.66 (0.28)	0.62 (0.30)	
EQ VAS (0–100)				0.49^a^
	mean (SD)	46.07 (19.67)	44.65 (18.5)	
PHQ-9 at baseline (0–27)				0.39^a^
	mean (SD)	13.77 (3.2)	13.48 (3.3)	

^a^Linear regression

^b^Logistic regression

CCSC: Canadian Cardiovascular Society Criteria; PHQ-9: Patient Health Questionnaire 9; SD: Standard deviation

### Base case analysis

#### Costs during follow-up

We found no statistically significant differences in unadjusted costs between IG and CG in the time periods from baseline to T1, from T1 to T2 and over the complete study period of 6 months. Yet, participants in the IG tended to have lower costs for inpatient services, outpatient physician services, formal nursing care and informal care than participants in the CG. The costs for medication and indirect costs tended to be higher in the IG. Unadjusted direct costs and total costs tended to be lower in the IG group (Table 3).

**Table 3:** Unadjusted costs, health-related quality of life and QALY after 1 month, after months 2–5 and after 6 months follow-up.

Cost category		Month 1 (mean (SD))		Months 2–6 (mean (SD))		6 Months (mean (SD))	
		Intervention group	Control group	Intervention group	Control group	Intervention group	Control group
Direct costs [€]		2,346 (3,519)	3,015 (4,403)	5,633 (10,807)	7,544 (13,426)	7,985 (12,185)	10,561 (15,130)
	Inpatient services [€]	1,382 (2,721)	1,833 (3,348)	3,404 (8,867)	4,224 (9,235)	4,789 (9,748)	6,059 (10,442)
	Outpatient physician services [€]	226 (218)	260 (265)	504 (600)	605 (923)	730 (700)	866 (1,029)
	Outpatient psychotherapist services [€]	27 (147)	20 (92)	51 (207)	60 (243)	79 (279)	81 (306)
	Outpatient non-physician services [€]	56 (111)	53 (118)	200 (434)	223 (574)	256 (484)	276 (625)
	Formal nursing care [€]	111 (762)	121 (738)	100 (750)	194 (1,776)	210 (1,155)	315 (2,017)
	Informal care [€]	298 (1,038)	508 (1,678)	697 (3,697)	1,714 (6,814)	996 (4,051)	2,222 (7,373)
	Medication [€]	245 (724)	221 (477)	674 (1,499)	521 (960)	920 (2,035)	743 (1,254)
Indirect costs [€]		303 (1,107)	204 (881)	1,189 (4,759)	424 (2,174)	1,490 (5,171)	628 (2,508)
Total costs [€]		2,649 (3,702)	3,219 (4,565)	6,826 (11,711)	7,971 (13,599)	9,479 (13,115)	11,192 (15,338)
QALY		0.0558 (0.0206)	0.0524 (0.0225)	0.2802 (0.1090)	0.2665 (0.1209)	0.3360 (0.1241)	0.3190 (0.1368)
ICER						Intervention dominates	

Differences between groups were tested by linear ordinary least squares regression; ICER: Incremental cost-effectiveness ratio; QALY: Quality adjusted life years; SD: Standard deviation;

After adjusting the costs for age, gender, baseline HRQL, baseline disease severities of angina pectoris and depression, and perceived mental illness stigma at baseline the direction of cost differences persisted. None of the differences were statistically significant (Table 4). Mean total costs were 1,309€ lower in the IG than in the CG.

**Table 4:** Differences in costs and QALY during 6 months follow-up.

Cost category		Adjusted difference between intervention and control group (SE)
Direct costs [€]		-2,098 (1,717)
	Inpatient services [€]	-1,033 (1,269)
	Outpatient physician services [€]	-104 (113)
	Outpatient psychotherapist services [€]	-6 (35)
	Outpatient non-physician services [€]	-10 (68)
	Formal nursing care [€]	-73 (207)
	Informal care [€]	-1,067 (779)
	Medication [€]	194 (193)
Indirect costs [€]		789 (429)
Total costs [€]		-1,309 (1,790)
QALY		0.0067 QALY (0.0133)
ICER		Intervention dominant

Adjustment for age, gender, years of schooling, symptom severity of angina pectoris at baseline, baseline HRQL and the perceived mental illness stigma at baseline was performed by linear ordinary least squares regression; ICER: incremental cost-effectivness ratio; QALY: quality adjusted life year; SE: standard error

#### Survival, quality of life and QALY during follow-up

11 patients died between T1 and T2 (IG: 4; CG: 7). As the exact date of death was not retrievable for all patients we decided to treat these participants as deceased at T1. In both groups the unadjusted HRQL measured by the EQ-5D index increased between baseline and T2 (Table 3). Differences between groups were not statistically significant.

During the study period of six months the IG experienced 0.3360 (SD: 0.1241) unadjusted QALY and the CG 0.3190 (SD: 0.1368), with no statistically significant difference between IG ad CG. After adjustment, the difference in QALY between IG and CG was 0.0067 QALY (SE: 0.0133) which was not statistically significant.

#### Cost-effectiveness

**Fig 2:**
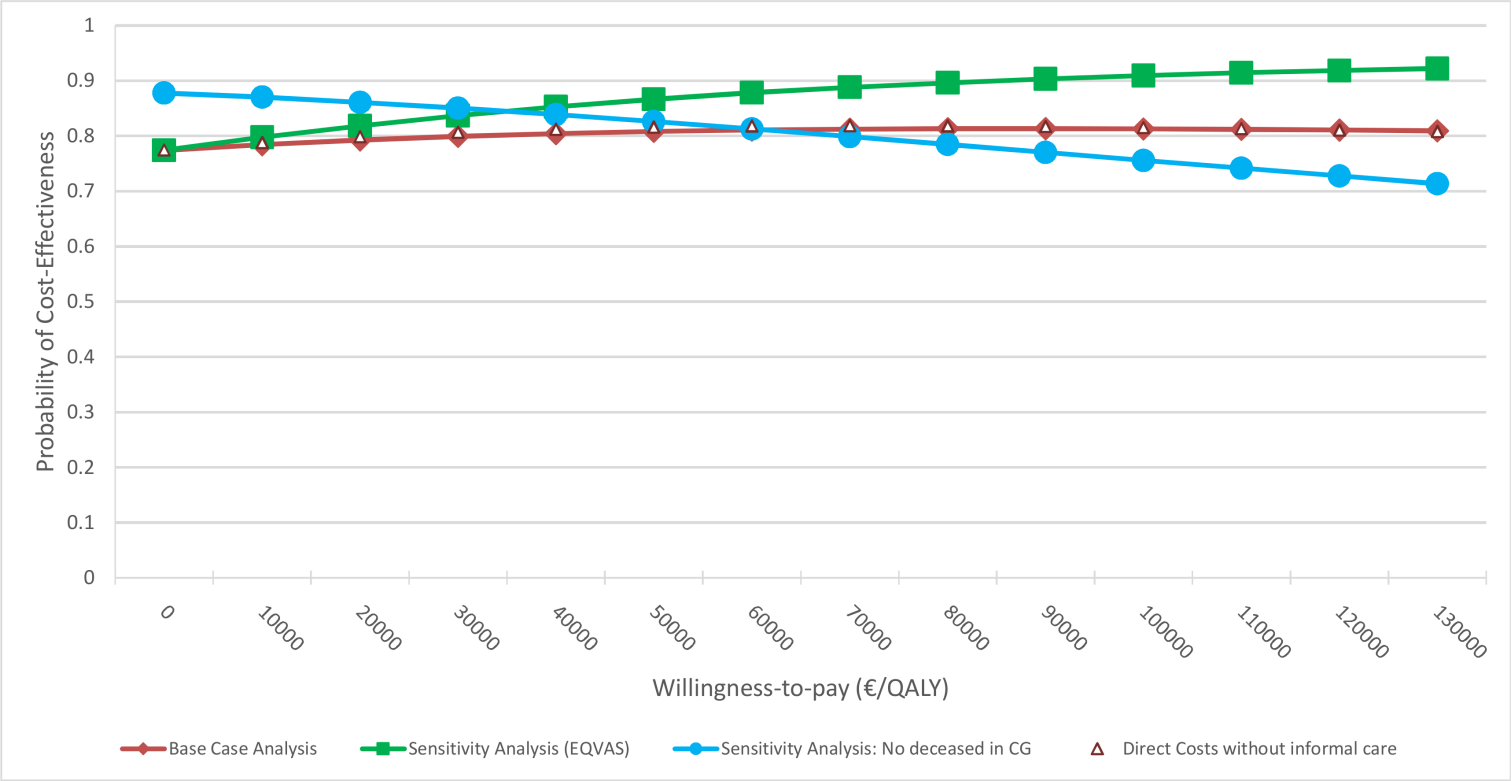
Cost-effectiveness acceptability curves of the base case and sensitivity analyses.

Over the complete study period of 6 months the ICER indicated dominance of the intervention. This means that the intervention was less expensive and more effective than the control treatment (Tables 3 and 4). The CEAC showed that the results were stable with regard to uncertainty in the data (Fig 2). At WTP margins of €0/QALY, €50,000/QALY and €130,000/QALY the probability of cost-effectiveness was around 80%.

### Sensitivity analysis

Based on the EQ VAS, participants in the IG gained 0.0135 QALY (SE: 0.0091) more than patients in the CG (Table 4). However, this result was not statistically significant. The ICER indicated dominance of the IG. The CEAC had an increasing trend with probabilities of cost-effectiveness ranging from 77% (at WTP €0/QALY) to 92% (at WTP €130,000/QALY) (Fig 2).

Considering all patients in the CG as survivors resulted in a QALY difference of -0.0049 (SE: 0.0128). The direction of the cost differences corresponded to the results of the base case analysis. Yet, the difference in total costs between the groups increased (€-2,078; SE: €1,848). All differences were not statistically significant. The probability of cost-effectiveness decreased with inceasing WTP (88% at a WTP of €0 per QALY to 71% at a WTP of €130.000 per QALY).

By choosing a payer’s perspective and including only direct costs without consideration of informal care the cost difference between IG and CG was comparable to the cost difference in the base case analysis (€-1,031; SE: €1,790; ns). The CEAC was nearly congruent with the CEAC of the base case analysis, i.e. the probability of cost-effectiveness at WTP margins of €0/QALY, €50,000/QALY and €130,000/QALY was around 80%.

The use of not adjusted QALY led to results similar to the base case analysis. The QALY difference between the groups decreased but was still in favour of the IG (0.0028; SE: 0.1278). The probability of cost-effectiveness decreased slightly between a WTP of €0/QALY (77%) and a WTP of €130,000/QALY (75%).

## Discussion

The results of our study provide arguments for the cost-effectiveness of a depression screening with patient-targeted feedback in cardiology. The intervention caused lower costs and better effects, with differences not being statistically significant though. However, in the probability for cost-effectiveness was constantly around 80% in the base case analysis, as indicated by the CEAC. This result was independent from the perspective of the analysis. The probability increased after changing the mode of QALY calculation. The assumption that all participants in the CG survived did not change the results significantly. In comparison to the base case analysis the probability of cost-effectiveness was higher at smaller WTP margins (€0 to €60,000/QALY) and smaller at higher WTP margins (€70,000 to €130,000/QALY).

The aim of the DEPSCREEN-INFO trial was to promote patient participation in the process of depression treatment and empower patients to seek support from professionals or informal sources like family or friends. We observed a noticeable reduction of direct costs in the IG. This was mainly attributable to the lower utilization of inpatient services and informal care. Furthermore, we observed an interesting difference in inpatient costs between IG and CG with respect to hospital type. The inpatient costs for care in a general hospital tended to be higher in the CG than in the IG (mean: €5,507 vs. €4,041; ns)). Contrary to this, inpatient costs for care in a psychiatric hospital tended to be higher in the IG than in the CG (mean: €860 vs €535; ns). This difference was more pronounced in the group of patients with severe depression, i.e. PHQ-score >14 (mean: €1227 vs €720; ns). Based on this we might assume that the intervention brought more patients into (adequate) depression treatment. This was also the case for psychotherapy. Patients with a severe depression in the IG tended to utilize more psychotherapy than patients in the CG (mean: €143 vs €89; ns). However, the potential of the intervention to bring patients into adequate treatment is limited. The German National Disease Management Guideline “Unipolar Depression” recommends, for example, psychotherapy as a treatment for patients with moderate and severe depression [40]. 63% of the patients in the IG suffered from moderate depression of whom only 10% were treated with psychotherapy within the study period; 37% of the patients in the IG suffered from a severe depression of whom only 19% received psychotherapy. This is an indicator of the limited impact of the intervention, which might be too weak to reach more patients. Considering the low threshold and intensity of the intervention this was to be expected. A further aspect to explain this observation could be the existence of a gap between depression recognition and treatment, which could be the result of long waiting times to access psychotherapy or the complexity of the interdisciplinary treatment of heart diseases with comorbid depression, which could prevent patients from seeking help on their own.

Furthermore, the intervention seems to reduce the need for informal and formal nursing care. Compared to the IG, mean costs for informal care in the CG were 70% higher (formal nursing care: 9%) at T1 and 145% higher (formal care: 94%) at T2, the differences not being significant though. This might be attributable to a more favourable trend in the development of depression as well as the heart disease (measured by the CCSC grade) in the IG.

As this is the first cost-effectiveness analysis of patient-targeted feedback in addition to depression screening in cardiology it is difficult to compare our results to the existing evidence. Previous cost-effectiveness analyses evaluated the cost-effectiveness of collaborative care in depression treatment of patients suffering from heart diseases [41–43]. In these studies cardiologic patients were screened for depression by means of a validated instrument (Beck Depression Inventory, PHQ-9, PHQ-2) and, if screened positive, enrolled into a collaborative care program. All studies concluded that the interventions were dominant, i.e. less costly and more effective. In our study, we also screened cardiologic patients and implemented a low-intensity intervention. We came up with similar results: the intervention tended to be cost-saving, not less effective and associated with a high probability of cost-effectiveness. We cannot conclude that our low-intensity intervention is as cost-effective as an elaborated collaborative care program. Yet, our results support the hypothesis that screening for depression is cost-effective. Given the small effort to implement this intervention, it represents a promising way to improve the health of the patient and to allocate the scare resources of the society.

Nevertheless, there are some limitations, which need to be discussed.

Firstly, there was no assessment of health care utilization at baseline. This means that we were not able to adjust for baseline costs in the regression models. Thus, it is possible that cost differences were biased. However, the groups were well balanced at baseline. Therefore, it is not very likely that cost differences at baseline were substantial enough to influence the costs at follow-up significantly.

Secondly, no participant died between baseline and T1. Eleven participants died between T1 and T2. As we had no precise dates of death we decided to treat them as deceased on the day of T1. This approach could have led to a bias in the results. To account for this, we considered all deceased patients in the CG as survivors in sensitivity analysis, which hardly changes the results.

## Conclusion

We were able to show that patient-targeted feedback in addition to depression screening in cardiology tends to be a cost-effective intervention. The intervention appears to bring more patients into depression treatment. Yet, this effect was limited. Nevertheless, the intervention is recommendable as it is easy and inexpensive to implement and bears positive effects for patients and society at reasonable costs.
